# Resilience and burnout status among nurses working in oncology

**DOI:** 10.1186/s12991-016-0121-3

**Published:** 2016-11-14

**Authors:** Sevinc Kutluturkan, Elif Sozeri, Nese Uysal, Figen Bay

**Affiliations:** 1 Department of Nursing, Gazi University Faculty of Health Sciences, Besevler, 06500 Ankara, Turkey; 2 Yıldırım Beyazıt Üniversity Faculty of Health Science, Ankara, Turkey; 3 Gazi University Health Research and Application Center, Gazi Hospital, Ankara, Turkey

**Keywords:** Burnout, Oncology nursing, Resilience

## Abstract

**Background:**

This study aimed to identify the resilience and burnout status of nurses working in the field of oncology.

**Methods:**

This descriptive study was conducted with 140 oncology nurses. The data were collected using a socio-demographic attributes form, Resilience Scale for Adults, and the Maslach’s Burnout Inventory. Percentage ratios, mean and median values, Kruskal–Wallis test, Mann–Whitney *U* test, correlation analysis, and multiple stepwise linear regression analysis were used to evaluate the data.

**Results:**

The Maslach’s Burnout Inventory total median score was 49.00. The emotional exhaustion median score was 24.00, the depersonalization median score was 9.00, and the personal accomplishment median score was 16.00. The Resilience Scale for Adults total median score was 134.00. The median resilience subscale scores, such as structural style, perception of future, family cohesion, self-perception, social competence, and social resources, were 16.00, 16.00, 24.00, 25, 23, and 31, respectively. A relationship existed between emotional exhaustion and perception of future; depersonalization and structured style and self-perception; and personal accomplishment and structured style, perception of future, and self-perception. Multiple stepwise linear regression analysis revealed a significant relationship between the number of years in the field and emotional exhaustion and depersonalization scores. Moreover, a significant relationship between structured style variables and personal accomplishment scores was observed.

**Conclusions:**

This study demonstrated the relationship between burnout and resilience situations among the oncology nurses. The results can be used to plan individual and organizational interventions to increase resilience and reduce the experience of burnout by developing measures such as improving communication skills, providing education on stress management and coping strategies, using social resources, and organizing programs that provide psychological support.

## Background

Burnout is an important problem frequently encountered in the scientific, social, and professional lives. One of the main factors that lead to burnout is exposure to stress for a long time. If the stress continues for a long time, the individual is negatively affected and experiences burnout. Some factors that lead to burnout in oncology include physical stressors (e.g., working under unsuitable conditions, long working hours, and insufficient tools and equipment as well as insufficient staff), psychological stressors (e.g., too many symptoms related to diseases and treatment, increased expectations of patients and families, and problems related to occupational safety), and administrational stressors (e.g., insufficient performance measures and unsatisfactory salaries) [1–3]. This burnout manifests in the form of emotional exhaustion, depersonalization, and a decrease in personal accomplishment. Emotional exhaustion represents the individual stress dimension of burnout. Depersonalization is the dimension where cold, uninterested, and strict and nonhuman attitudes develop toward the person’s job or toward other people from work-related relations. A diminished sense of personal accomplishment is the reduction in a person’s sense of competence and feelings of success [4]. In the study conducted by Trufelliet et al. [5] emotional exhaustion was 36%, depersonalization was 34%, and low personal accomplishment was 25% in oncology professionals.

Burnout has negative effects on physical, emotional, and mental health. One of the most important factors to prevent burnout is the effective management of the sources of stress which lead to burnout. Individuals’ personality traits and psychological functions are the most important factors in stress management and preventing burnout [6]. In recent years, the concept of psychological resilience has emerged as a personality trait that is protective against burnout [7–9]. Despite a number of descriptions focusing on different aspects of resilience, which has a multidimensional and learnable structure, resilience is defined as a person’s adaptation to important stressful sources such as trauma, threat, tragedy, familial and relationship problems, and workplace and financial issues [8, 10]. Friborg et al. [10] emphasized six factors to explain the structure of resilience. These factors are self-perception, perception of future, structured style, social competence, family cohesion, and social resources. Self-perception is the state of a person being aware of himself or herself. Perception of future is the individual’s perspective of the future. Structured style is the person’s personal attributes such as self-confidence, strengths, and self-discipline. Social competence is where persons are supported socially. Family cohesion is the individual’s harmony with those closest to them [10].

With the increase in resilience, the individual is able to cope with barriers, uncertainty, and many similar negative situations, and increase their ability to be successful. With the enhancement of resilience, the nurses are able to cope with the negative conditions better, their abilities of adaptation and achievement are increased, and they probably experience less burnout. The potential association between resilience and burnout in the oncology nurses is completely unexplored [9–11].

It is hypothesized that the presence of resilience in the oncology nurses might be associated with a lower prevalence of burnout. To test this hypothesis, a survey was conducted to determine (a) the burnout and resilience states, (b) the factors influencing burnout and resilience, and (c) the relationship between burnout and resilience in oncology nurses. Understanding the concept of resilience and burnout can assist in providing support and developing programs to help nurses become and stay resilient.

## Methods

### Study sample

This study was carried out with nurses actively working in the oncology–hematology clinic and the chemotherapy Administration Unit and Policlinic. The entire population was included in the study sample with no specific sample selection. Inclusion criteria for the study were experience in the oncology–hematology clinics and willingness to volunteer for the study. Exclusion criteria for the study were working in departments other than oncology–hematology clinic/polyclinic, not actively working as a nurse, and not accepting to participate in the study. Ten nurses who refused to participate and 20 nurses who did not complete the forms were not included in the study. The study was carried out with 140 oncology nurses.

### Procedure and measures

The data for this study were collected using a socio-demographic attributes form, Resilience Scale for Adults, and Maslach’s Burnout Inventory.

#### Socio-demographic attributes form

The socio-demographic attributes form contained two different sections. The first section contained socio-demographic features (age, sex, marital status, educational status, dependents they care for, family type, and the existence and the number of children), and the second section contained career attributes (years of professional experience, duty, and working hours).

#### Maslach’s Burnout Inventory

The Maslach’s Burnout Inventory was created by Maslach and Jackson in 1981. The inventory contained 22 items and 3 subscales of emotional exhaustion, depersonalization, and personal accomplishment. In the emotional exhaustion subscale, eight items were related to fatigue, being fed up, and the reduction of emotion energy. In the depersonalization subscale, six items were about the individual’s behaviors that lacked emotion toward those who were cared for and were given service to. In the personal accomplishment dimension, eight items defined the situation where the person felt sufficient and successful [12].

The Maslach’s Burnout Inventory was evaluated according to a 5-point Likert scale where 0 points denotes “never” and 4 points denotes “always.” For this study, the minimum scores on the sub-dimensions were subtracted from the maximum scores, and then the scores were divided by three, which gave the cutoff points. It was expected that in individuals experiencing burnout, the emotional exhaustion (30 and above: high; 19–29: moderate; 8–18; low) and depersonalization scores (23 and above: high; 15–22: moderate; 6–14: low) would be high and the personal accomplishment scores (30 and above: high; 19–29: moderate; 8–18: low) would be lower.

The Inventory’s Turkish reliability and validity study was conducted by Cam [13] and Ergin [14]. Cronbach’s alpha coefficient was found to be 0.83 for emotional exhaustion, 0.71 for depersonalization, and 0.72 for personal accomplishment [13, 14]. In this study, Cronbach’s alpha coefficient was 0.70 for emotional exhaustion, 0.78 for depersonalization, and 0.76 for personal accomplishment. The total score was 0.78.

#### Resilience Scale for Adults

The Resilience Scale for Adults was created by Friborg et al. [10]. The scale contained 33 items and 6 subscales measuring self-perception, perception of future, structured style, social competence, family cohesion, and social resources. In measuring resilience as high or low, scoring was left free. When scores on the scale increased and resilience was desired to increase, then from left to right, the answer boxes were evaluated as 1, 2, 3, 4, and 5. If the scores decreased and resilience was desired to increase, then the answer boxes were evaluated as 5, 4, 3, 2, and 1. The total score from the inventory was then divided into the number of items, and the median scores were evaluated. High scores obtained on the inventory indicate high resilience scores [10]. The reliability and validity study for the Turkish scale was conducted by Basım and Cetin [8]. Cronbach’s alpha coefficients for the subscales were found to be between 0.66 and 0.81 [8]. In the present study, Cronbach’s alpha coefficient was 0.71 for structural style, 0.71 for perception of future, 0.70 for self-perception, 0.64 for family cohesion, 0.70 for social competence, and 0.70 for social resources. The total score was 0.73.

### Statistics

The data collected for this study were analyzed by the researchers using Statistical Package for Social Science (SPSS 21) for Windows package program. Demographic information for the nurses was reported as frequencies and percentages. Continuous variables were reported as mean ± standard deviation or median and interquartile range (IQR) as appropriate. Normality of the Maslach’s Burnout Inventory and Resilience Scale for Adults scores were examined by the Shapiro–Wilk test. Scale scores were not normally distributed, and nonparametric tests were used. To examine the relationship between the Burnout Inventory subscales, the Resilience Scale for Adults subscales, and the socio-demographic attributes, the Mann–Whitney *U* (MU) test and the Kruskal–Wallis (KW) test were used. To examine whether a significant relationship existed between the Maslach’s Burnout Inventory’s subscales and the Resilience for Adults subscales, Spearman’s correlation coefficient was evaluated. Central limit theorem was based on the regression analysis, although data source was not normally distributed. According to the law of large numbers, *n* → ∞ the basis of knowledge of the distribution of the sample mean for normal distribution to approximate regression analysis was performed. Multiple stepwise linear regression analysis was conducted to explore the factors affecting the burnout. Cronbach’s alpha was computed for the Maslach’s Burnout Inventory and Resilience Scale for Adults instruments to assess the internal reliability of the questions. The level of significance was determined after the pairwise comparison Bonferroni correction.

## Results

Nurses’ socio-demographic and career attributes data are shown in Table 1.

**Table 1:** Nurses’ socio-demographic and career attributes

Socio-demographic characteristics	Number	%
Sex		
Female	126	90
Male	14	10
Age groups (years)		
19–27	23	16.2
28–35	55	39.4
36–44	49	35
45–53	13	9.4
Marital status		
Married	85	59.3
Single	55	40.7
Education status		
High school graduate	21	15
Associate’s degree	27	19.3
Bachelor’s degree	84	60
Higher education graduate	8	5.7
Family type		
Nuclear	126	90
Extended	11	7.9
Divorced	3	2.1
Any children		
Yes	78	56.1
No	62	43.9
Dependents		
Yes	73	52.5
No	67	47.5
People taken care of		
Child	45	61.6
Parents	21	28.8
Sibling	7	9.6
Number of children		
1	34	46.6
2	35	48
3 or more	4	5.4
Career attributes	Number	%
Number of years in the field (years)		
1–8	56	40
9–16	44	31.4
17–24	31	22.2
25–33	9	6.4
Number of years working in oncology	Mean ± SD 3.70 ± 3.69	
Clinic stationed at		
Oncology day treatment	68	48.6
Oncology/medical oncology clinic	48	34.3
Hematology clinic	12	8.6
Bone marrow transplantation clinic	6	4.3
Palliative care clinic	2	1.4
Internal medicine clinic	2	1.4
Hematology day treatment	2	1.4
Duty at current clinic		
Clinical nurse	84	60
Chief nurse	40	28.5
Policlinic nurse	16	11.5
Working hours at the current clinic		
8–16	77	55
8–16 or 16–24	36	25.7
16–08 or 16–24	18	12.9
16–24 or 24–08	9	6.4
Previous clinics of practice		
Oncology/hematology clinics	91	34.9
Internal medicine clinics	68	26.1
Surgical clinics	47	18
Intensive care clinics	32	12.2
Pediatric clinics	16	6.1
Gynecology clinics	7	2.7

### Maslach’s Burnout Inventory results

The total median score and interquartile range for Maslach’s Burnout Inventory was 49.00: emotional exhaustion (EE), depersonalization (D), and personal accomplishment (PA) median scores of the oncology nurses were found to be 24.00, 9.00, and 16.00 respectively.

According to the nurses’ demographic information, as per the median score distribution of the Maslach’s Burnout Inventory, the nurses who did not have any dependents and worked in the field between 1 and 8 years had higher emotional exhaustion median scores and the difference between the median scores was found to be statistically significant (*p* < 0.05) (Table 2).

**Table 2:** Distribution of the Maslach’s Burnout Inventory scores of oncology nurses according to their socio-demographic characteristics and career attributes

	Maslach’s Burnout Inventory		
	Emotional exhaustion (median, IQR)	Depersonalization (median, IQR)	Personal accomplishment (median, IQR)
Dependents			
Yes	23.00 (20.0;28.0)	8.00 (5.0;10.0)	16.00 (12.2;19.0)
No	26.00 (21.0; 31.0)	10.00 (7.0;13.0)	16.00 (13.0;20.0)
	MU = 1951,000, *p* = 0.039*	MU = 1700,000, *p* = 0.002*	MU = 2366,500, *p* = 0.857
Years of professional experience (years)			
1–8	25.50 (22.0; 31.0)	9.50 (7.0; 13.0)	17.00 (13.0; 20.0)
9–16	24.00 (20.0; 29.7)	9.00 (6.0; 11.0)	16.00 (13.0; 18.0)
17–24	23.00 (19.0; 29.0)	8.00 (5.0; 11.0)	16.00 (12.0; 20.0)
25–33	20.00 (16.5; 22.5)	8.00 (5.5; 9.0)	14.00 (12.0; 17.0)
	KW = 9.841, *p* = 0.020*	KW = 5.294, *p* = 0.151	KW = 2.159, *p* = 0.540

* *p* < 0.05

### Resilience Scale for Adults results

The total median score and interquartile range for Resilience Scale for Adults was 134.00 (122.0; 146.0). The median scores of the nurses for structural style, perception of future, family cohesion, self-perception, social competence, and social resources were found to be 16.00, 16.00, 24.00, 25.00, 23.00, and 31.00 respectively.

The median score distribution for the Resilience Scale for Adults by nurses’ demographic characteristics indicated that the nurses between the ages of 36 and 44 years had higher structured style and self-perception median scores compared with the other age groups, and the difference was statistically significant (*p* < 0.05).

The nurses who had children compared with the nurses who did not have children had higher self-perception median scores, and the difference between the median scores was statistically significant (*p* < 0.05) (Table 3).

**Table 3:** Distribution of the Resilience Scale for Adults scores of oncology nurses according to their socio-demographic characteristics and career attributes

	Resilience Scale for Adults					
	Structural style (median, IQR)	Perception of future (median, IQR)	Family cohesion (median, IQR)	Perception of self (median, IQR)	Social competence (median, IQR)	Social resources (median, IQR)
Age groups (years)						
19–27	17.00 (12.0;18.0)	17.00 (15.0; 20.0)	24.00 (20.0; 26.0)	24.00 (20.0; 26.0)	22.00 (19.0; 24.0)	29.00 (27.0; 32.0)
28–35	15.00 (13.0; 16.0)	15.00 (12.0; 19.0)	23.00 (20.0; 26.0)	24.00 (21.0; 27.0)	23.00 (20.0; 26.0)	31.00 (28.0; 34.0)
36–44	16.00 (15.0; 19.0)	16.00 (14.0; 19.0)	25.00 (21.0; 28.0)	26.00 (22.0; 28.0)	22.00 (20.0; 28.0)	31.00 (29.0; 33.0)
45–53	16.00 (14.0; 20.0)	17.00 (15.0; 19.5)	25.00 (21.0; 27.0)	26.00 (23.0; 29.5)	26.00 (23.0; 30.0)	32.00 (29.0; 35.0)
	KW = 12.268, *p* = 0.007*	KW = 3.854, *p* = 0.278	KW = 2.075, *p* = 0.557	KW = 8.008, *p* = 0.046*	KW = 4.938, *p* = 0.176	KW = 4.930, *p* = 0.177
Education status						
High school	16.00 (14.0; 18.5)	17.00 (14.5; 20.0)	25.00 (22.0; 28.0)	24.00 (22.0; 28.5)	22.00 (20.0; 27.0)	29.00 (27.0; 32.0)
Associate’s degree	16.00 (15.0; 20.0)	16.00 (14.0; 20.0)	25.00 (22.0; 27.0)	26.00 (24.0; 29.0)	24.00 (20.0; 29.0)	32.00 (31.0; 35.0)
Bachelor’s degree	16.00 (13.0; 18.0)	16.00 (13.2; 19.0)	23.00 (20.0; 26.7)	24.00 (21.0; 27.0)	23.00 (20.0; 26.7)	31.00 (28.0; 34.0)
Higher education	15.00 (12.0; 18.0)	15.00 (14.0; 17.0)	23.00 (20.0; 26.0)	21.00 (19.0; 26.2)	21.00 (19.0; 24.0)	30.00 (28.0; 33.0)
	KW = 2.733, *p* = 0.435	KW = 1.577, *p* = 0.665	KW = 3.632, *p* = 0.304	KW = 6.462, *p* = 0.091	KW = 2.726, *p* = 0.436	KW = 10.045, *p* = 0.018*
Have children						
Yes	16.00 (14.0; 18.5)	16.00 (14.0; 19.0)	25.00 (21.5.0; 27.0)	26.00 (22.0; 28.0)	23.00 (20.5; 27.5)	31.00 (29.0; 34.0)
No	15.50 (12.7; 17.7)	16.00 (13.0; 19.0)	23.00 (20.0; 26.0)	24.00 (21.0; 26.5)	22.00 (19.0; 26.0)	31.00 (28.0; 33.0)
	MU = 2054,000, *p* = 0.155	MU = 2321,50, *p* = 0.780	MU = 1980,50, *p* = 0.084	MU = 1880,00, *p* = 0.044*	MU = 2029,50, *p* = 0.129	MU = 2218,50, *p* = 0.472
Number of years in the field (years)						
1–8	16.00 (13.0; 17.0)	16.00 (13.0; 19.0)	24.00 (21.0; 26.0)	23.00 (20.2; 26.0)	22.00 (19.0; 25.7)	30.50 (22.2; 33.0)
9–16	15.00 (13.0; 17.0)	16.00 (13.0; 18.0)	23.50 (20.0; 27.0)	25.00 (21.0; 28.0)	22.00 (20.0; 26.0)	31.00 (28.0; 34.70)
17–24	18.00 (15.0; 20.0)	16.00 (14.0; 20.0)	24.00 (20.0; 27.0)	26.00 (24.0; 29.0)	25.00 (21.0; 28.0)	31.00 (30.0; 33.0)
25–33	18.00 (14.0; 20.0)	19.00 (15.5; 20.0)	25.00 (21.0; 28.5)	29.00 (24.5; 30.0)	26.00 (22.0; 30.0)	32.00 (28.0; 35.0)
	KW = 11.535, *p* = 0.009*	KW = 5.421, *p* = 0.143	KW = 0.773, *p* = 0.856	KW = 13.056, *p* = 0.005*	KW = 7.563, *p* = 0.056	KW = 2.936, *p* = 0.402

* *p* < 0.05

The results of the analyses conducted with the other socio-demographic variables (gender, age, marital status, education level, family type, presence of any children, length of service in oncology, position and working hours) did not reveal any statistically significant differences (*p* > 0.05).

The median scores for the Resilience Scale for Adults and the nurses’ education level suggested that the social resources’ median scores were significantly higher for the nurses who had an associate’s degree (Table 3).

A statistically significant difference was reported between the median scores of structured style and self-perception based on the number of years the nurses had worked in the field. The significance tests conducted for multiple variables revealed a significant difference between the nurses working in the field for 17–24 years and the nurses working in the field for 1–8 years (*p* < 0.05) (Table 3). The results of the analyses conducted with the other socio-demographic variables (gender, marital status, family type, dependents they care for, length of service, position and working hours) did not reveal any statistically significant differences (*p* > 0.05).

### Resilience Scale for Adults and Maslach’s Burnout Inventory results

Spearman’s correlation analysis of the median scores for the Resilience Scale for Adults and the Maslach’s Burnout Inventory suggested a significantly negative correlation between emotional exhaustion and perception of future; depersonalization and structured style and self-perception; and personal accomplishment and structured style, perception of future, and self-perception (Table 4). The correlation analysis between the total scale scores showed that there was a negative and significant correlation.

**Table 4:** Spearman’s correlation between the Resilience Scale for adults and the Maslach’s Burnout Inventory median scores

Resilience Scale for Adults subscales	Maslach’s Burnout Inventory subscales			Total
	Emotional exhaustion	Depersonalization	Personal accomplishment	
Structural style	−.107	−.195*	−.300*	−.258*
Perception of future	−.222*	−.142	−.272*	−.287*
Family cohesion	0.029	−.049	−.095	−.045
Perception of self	−.*226*	−.210*	−.452*	−.394*
Social competence	−.108	−.092	−.130	−.135
Social resources	−.013	−.072	−.155	−.107
Total	−.191*	−.161	−.350*	−.320*

* *p* < 0.01

Multiple regression analysis revealed a significant correlation between the number of years in the field, emotional exhaustion scores, and depersonalization (*p* < 0.01). This variable explained 4.97 and 3.49% of the total variance, respectively. A significant correlation was observed between structured style and personal accomplishment scores. This variable explained approximately 6.12% of the total variance (Table 5).

**Table 5:** Results of regression analysis of the effect of independent variables on burnout

Independent variables	Dependent variable	Beta	Standard error	R2	Standardize beta	*t*	*p*
Number of years in the field	EE	−1.792	0.0619	0.049	−.236	−.2.846	*0.000*
Number of years in the field	D	−.748	0.308	0.034	−.203	−2.426	*0.017*
Structural style	PA	−.497	0.156	0.062	−.262	−3.179	*0.002*

Italic values indicate significance of *p* value (*p* < 0.05)

*EE* emotional exhaustion, *D* depersonalization, *PA* personal achievement

## Discussion

This section will be presented in three parts based on the findings of this study. The first part presents the status of having burnout and the variables that are influential on burnout. The second one is the state of resilience and the variables that are influential on resilience. The third one is the dimension of the correlation between burnout and resilience and the factors that are influential on this correlation.

### Burnout

The total median score and interquartile range for Maslach’s Burnout Inventory was 49.00 (43.0; 59.0). Many factors are effective in the manifestation of burnout. The literature reports a negative relationship between the level of burnout and the age, working time, and field experience [15–17]. While some factors have an effect on emotional exhaustion, other factors have an effect on depersonalization and personal accomplishment. An important fact related to emotional exhaustion is the time working in the field. Individuals with little field experience, wanting to be recognized in the field in a short period of time, believing that they will earn back all of their efforts very quickly, and experiencing disappointments when they do not reach their goals may emotionally burn out much faster [16–18]. In this study, emotional exhaustion was experienced much more, especially in nurses with less experience in the field. Similarly, the regression analysis revealed a negative correlation between the number of working years in the field and emotional exhaustion and depersonalization.

In this study, having dependents to care for as a socio-demographic variable was influential in oncology nurses experiencing emotional exhaustion. Akyüz [19] reported results similar to those observed in this study where individuals having dependents to care for had higher ratios of emotional exhaustion and depersonalization. The role of a caregiver leads to individuals experiencing emotional exhaustion more frequently and, as a result, increases the rate of developing burnout [20, 21]. Demir et al. [15] found that continuous day work reduced depersonalization, and the nurses in charge had high personal accomplishment levels. This study also found a negative correlation between working status and depersonalization.

### Resilience

Resilience is the person’s ability to successfully cope with barriers, uncertainty, and similar negative situations [22]. In this study, in terms of resilience, oncology nurses’ scores on structured style, family cohesion and social competence, perception of future, self-perception, and social resource were close to the expected level. A person’s resilience level is affected by individual, familial, and environmental factors. Individual factors such as age either positively or negatively influence the individual’s psychological development starting from childhood and continuing through adulthood [23]. In this study, the oncology nurses in the age group of 36–44 years had higher median scores for structured style and self-perception. Regarding the reasons for the relationship between age and structured style, which represents the person’s strengths, it is thought that the adults in the age group of 36–44 years are in a period where they know themselves better and have more self-confidence. In this study, one factor affecting resilience in the oncology nurses was the number of working years. Demir et al. [15] found that the personal accomplishments increased with the number of working years. Finn [24] reported that as the nurses’ field experience increased, they had stronger professional autonomy and higher job satisfaction.

Having children was another factor affecting resilience. In a study performed on radiation therapists and oncology nurses, 53.6% had children and their resilience levels were reported to be moderate [25]. In this study, nurses who had children had higher median scores for resilience indicator of self-perception.

Another factor affecting the oncology nurses’ resilience was the level of education. In this study, compared with other nurses, nurses with an associate’s degree had better levels of resilience, as they were thought to be using social resources in a much better way. The use of social resources is closely related to the education level. As the education level increases, the individuals realize the importance of social support resources, learn how to access resources, and increase their use [24]. One of the coping methods doctors and nurses, working with cancer patients, use to cope with work-related stresses is the use of social support [26]. Lim et al. [27] reported that nurses used spouse, friend, and family support as a coping strategy for stress. In a study conducted on pediatric oncology nurses aimed at determining their coping and resilience, it was found that to cope and increase their resilience, and the nurses needed to use their social support resources very well [28].

The number of working years in the field is another variable that influences resilience. As the number of working years increases, the oncology nurses’ ability to cope with stress also increases and they become aware of themselves. A study conducted on health care professionals working with cancer patients reported that the nurses working between 1 and 10 years had higher stress scores compared with the nurses working for 11 years or more [26]. In this study, the nurses working in the field for 17–24 years had higher median scores for structured style and self-perception compared with the nurses working for 1–8 years. Another study reported that age, professional experience, education, and the number of working years had no effect on resilience [29]. It is estimated that as the nurses’ professional experience increases, their self-confidence increases too, and they become more aware of their competence/lack of competence.

### Burnout and resilience

Resilience, in general, refers to a success or adaptation period [8]. Burnout and resilience, which are influenced by personal and professional factors, are often seen when adaptation is not possible. Personal and professional factors can lead to the stress factors causing burnout. A person’s self-perception is among the personal factors. If the person’s self-awareness is low, their confidence that they will accomplish good things is also low. They exaggerate barriers and give up fighting with the barriers very quickly. The person focuses on their failures and not their successes, and their susceptibility to burnout increases [30, 31]. This study reported a negative correlation between the subscales of emotional exhaustion and self-perception. It has been noted that individuals with insufficient self-competence constitute high-risk groups in terms of burnout [32, 33]. Garrosa et al. [34] reported a negative relationship between the nurses’ personal resources and control situations where they experience emotional exhaustion.

Having negative expections and perceptions about the future leads to burnout. Taorimo and Law [35] reported that burnout was significantly affected by perceptions of the future. Similarly, individuals experiencing burnout have not been very successful in the past and, with this perspective, assume that they will not be successful in the future either [36]. Coping with stress allows the person to know themselves better and, by establishing positive expectations regarding the future, their resilience increases and their burnout is better controlled. This study found a significant negative relationship between emotional exhaustion and perception of future.

In this study, personal accomplishments influenced structured style, perception of future, and self-perception. Moreover, the regression analysis indicated a relationship between the structured style, which represented the strong sides of a person, and personal accomplishment. While increasing resilience increases personal accomplishments, burnout reduces a person’s personal accomplishments. A decrease in the feeling of personal success manifests when a person sees their job performance as weak and evaluates themselves negatively. When people feel insufficient and unsuccessful, it causes them to lose their self-respect. The person may think that their contributions to work and society are limited [17, 37]. A study conducted with health care personnel working in an oncology center reported that doctors had much higher levels of emotional exhaustion and depersonalization and nurses lacked much more in personal success [38].

## Conclusions

This study demonstrated the relationship between burnout and resilience situations among the oncology nurses. It was found that to increase their resilience, the nurses should be supported in structured style, perception of future, and perception of self. For less experience of burnout, they should not experience emotional exhaustion and should increase their personal accomplishments. Hence, the results of this study can be used to plan individual and organizational interventions to increase resilience and reduce the experience of burnout by developing measures such as improving communication skills, providing education on stress management, organizing programs that provide psychological support, using psychodrama and relaxation techniques, establishing a positive work environment, and so forth.

This study had some limitations. The results of this study were limited by the small sample size. Also, the nurses were evaluated for burnout and resilience only once. However, the study reflected what nurses working in an oncology clinic actually experience. Another important limitation is that 20 nurses were excluded from the analysis part of the study since there was some missing information in their research forms. Statistical data analysis was performed on 140 nurses with no missing information.

## Abbreviations

D: depersonalization
EE: emotional exhaustion
IQR: interquartile range
KW: Kruskal–Wallis
MU: Mann–Whitney *U*
PA: personal achievement
